# Silencing of Vangl2 attenuates the inflammation promoted by Wnt5a via MAPK and NF-κB pathway in chondrocytes

**DOI:** 10.1186/s13018-021-02268-x

**Published:** 2021-02-15

**Authors:** Ke Zhang, Zhuoying Li, Yunyang Lu, Linyi Xiang, Jiadong Sun, Hong Zhang

**Affiliations:** 1 Hospital of Stomatology, Sun Yat-sen University, Guangdong Provincial Key Laboratory of Stomatology, Guanghua School of Stomatology, Sun Yat-sen University, No.56 Lingyuan West Road, Guangzhou 510055 Guangdong, People’s Republic of China; 2 Department of Stomatology, Binhaiwan Central Hospital of Dongguan (also called The Fifth People’s Hospital of Dongguan), The Dongguan Affiliated Hospital of Medical College of Jinan University, No.111 Humen Road, Humen Town, Dongguan City, 523905 Guangdong Province People’s Republic of China

**Keywords:** Chondrocytes, Osteoarthritis, Vangl2, Collagen, MMPs, Wnt5a/PCP

## Abstract

**Background:**

The Wnt planar cell polarity (PCP) pathway is implicated in osteoarthritis (OA) both in animals and in humans. Van Gogh-like 2 (Vangl2) is a key PCP protein that is required for the orientation and alignment of chondrocytes in the growth plate. However, its functional roles in OA still remain undefined. Here, we explored the effects of Vangl2 on OA chondrocyte in vitro and further elucidated the molecular mechanism of silencing Vangl2 in Wnt5a-overexpressing OA chondrocytes.

**Methods:**

Chondrocytes were treated with IL-1β (10 ng/mL) to simulate the inflammatory microenvironment of OA. The expression levels of Vangl2, Wnt5a, MMPs, and related proinflammatory cytokines were measured by RT-qPCR. Small interfering RNA (siRNA) of Vangl2 and the plasmid targeting Wnt5a were constructed and transfected into ATDC5 cells. Then, the functional roles of silencing Vangl2 in the OA chondrocytes were investigated by Western blotting, RT-qPCR, and immunocytochemistry (ICC). Transfected OA chondrocytes were subjected to Western blotting to analyze the relationship between Vangl2 and related signaling pathways.

**Results:**

IL-1β induced the production of Vangl2, Wnt5a, and MMPs in a time-dependent manner and the significantly increased expression of Vangl2. Vangl2 silencing effectively suppressed the expression of MMP3, MMP9, MMP13, and IL-6 at both gene and protein levels and upregulated the expression of type II collagen and aggrecan. Moreover, knockdown of Vangl2 inhibited the phosphorylation of MAPK signaling molecules (P38, ERK, and JNK) and P65 in Wnt5a-overexpressing OA chondrocytes.

**Conclusions:**

For the first time, we demonstrate that Vangl2 is involved in the OA process. Vangl2 silencing can notably alleviate OA progression in vitro by inhibiting the expression of MMPs and increasing the formation of the cartilage matrix and can inhibit the proinflammatory effects of Wnt5a via MAPK and NF-κB pathway. This study provides new insight into the mechanism of cartilage inflammation.

## Introduction

Osteoarthritis (OA) is the most prevalent joint disorder characterized by chronic inflammation, progressive destruction of articular cartilage, and subchondral bone sclerosis [1, 2]. The complex pathogenesis of OA involves mechanical, inflammatory, and metabolic factors, which ultimately lead to structural destruction of articular cartilage and failure of the synovial joint [3]. Chondrocytes are the only resident cells in the articular system and are critical for maintaining the dynamic equilibrium between anabolism and catabolism in the extracellular matrix (ECM) [2, 4]. Several risk factors such as abnormal mechanical stress and proinflammatory cytokines have been shown to reduce chondrocytes and degrade the ECM in cartilage [5, 6]. Although increasing efforts have been dedicated to revealing the pathological process of irreversible structural change, the molecular mechanisms underlying this disease still remain elusive.

OA is an active dynamic alteration, which arises from an imbalance between the repair and destruction of joint tissue, regulated by a complex network of different molecular pathways, including the Wnt signaling network [7, 8]. Wnt5a, a representative of the Wnt family, activates the non-canonical Wnt pathway which includes the planar cell polarity (PCP) pathway and the Ca2+ pathway. Wnt5a has been shown to play a critical biological role in cartilage inflammation as it promotes the catabolic signaling of chondrocytes [9]. Increased Wnt5a signaling in OA has been implicated in the upregulation of inflammatory cytokines, chemokines, and matrix metalloproteinases (MMPs) as well as reduction of type II collagen (Col-2) and aggrecan [9–11].

Van Gogh-like 2 (Vangl2) is a core PCP component that regulates Wnt/PCP signaling [12–15]. Recently, evidence has accumulated that the pathogenesis of OA is associated with abnormal activation of Wnt/PCP pathway, which regulates the expression of MMPs and cartilage-related genes [9, 10, 16–18]. Notably, previous research suggested that Vangl2 plays a crucial role in cartilage development and disease. An in vitro study revealed that Vangl2 participates in Wnt5a/PCP signaling that promotes the formation of organized columns of isolated growth plate chondrocytes [19]. Animal experiments also indicate that during skeletal development, Wnt5a-induced Vangl2 phosphorylation facilitates growth plate chondrocyte columnar organization and limb growth [20, 21]. In addition, mutated Vangl2 in mice results in shortened limbs and reduced bone volume [21–23]. However, despite these findings, our understanding of the precise role of Vangl2 in OA chondrocytes is limited. In this study, we demonstrate for the first time that there is a link between Vangl2 and OA process and investigate the potential molecular mechanisms.

## Materials and methods

### Chondrocytes culture

ATDC5 cells (Fuheng Centre Cell Bank, China), a murine chondrogenic cell line, were cultured in Dulbecco’s modified Eagle’s medium/Ham’s F-12 nutrient mixture (DMEM/F-12; Gibco, USA) containing 10% fetal bovine serum (FBS; Gibco) and 1% penicillin/streptomycin (Gibco, USA). Cells were maintained in a humidified atmosphere containing 5% CO2 at 37 °C. After the cells had reached 80–90% confluence, they were collected for experimentation. The medium was changed every 2 days. Then, ATDC5 were treated with recombinant mouse IL-1β (10 ng/mL) to simulate the inflammatory environment of OA.

### Cell transfection

Chondrocytes were inoculated into 6-well plates (Corning, NY, USA) at a density of 5 × 10^5^ cells/well (Corning, NY, USA). According to the manufacturer’s instructions, the Vangl2 small interfering RNA (siRNA; RiboBio; Guangzhou, China) and negative control (nc; RiboBio; Guangzhou, China) were transfected by using Lipofectamine RNAiMAX (Invitrogen, CA, USA) when the cell fusion rate reached 50–70%. To establish Wnt5a overexpressing cells, we used Lipofectamine 2000 (Invitrogen, CA, USA) to transfect plasmids targeting Wnt5a and negative control (NC) after cells reached 70% confluence. The medium was replaced with DMEM/F12 supplemented with 10% FBS after 6 h.

### RNA quantification by real-time quantitative polymerase chain reaction (RT-qPCR) amplification

Total cell RNA was extracted by using TRIzol. The mRNA was mixed with the PrimeScript RT Master Mix (Perfect Real Time; Takara, Japan) and RNase-free water, then reverse-transcribed into cDNA according to the manufacturer’s instructions. The primers used for RT-qPCR were listed in Table 1. qPCR was performed by the SYBR® Green RT-qPCR kit (Roche Diagnostics, Switzerland) and a Roche light cycle 96 (Roche, Switzerland). The GAPDH mRNA expression level is used for normalization, and the mRNA expression levels were calculated using the 2^−ΔΔcq^ method. We assigned an arbitrary value of 1 for each control expression level. The treated samples were evaluated as fold change over control.

**Table 1:** Primer sequences for PCR

Gene (mouse)	Primer sequence (3′→5′) forward	Primer sequence (3′→5′) reverse
Wnt5a	ATGCAGTACATTGGAGAAGGTG	CGTCTCTCGGCTGCCTATTT
Vangl2	GGGATGGGAGTCGTGGAGATA	TCATGGGAGATACTGTGCTCAG
MMP3	ACATGGAGACTTTGTCCCTTTTG	TTGGCTGAGTGGTAGAGTCCC
MMP9	CTGGACAGCCAGACACTAAAG	CTCGCGGCAAGTCTTCAGAG
MMP13	CTATCCCTTGATGCCATTACCAG	ATCCACATGGTTGGGAAGTTC
Col-2	CAGGATGCCCGAAAATTAGGG	ACCACGATCACCTCTGGGT
IL-6	CCAAGAGGTGAGTGCTTCCC	CTGTTGTTCAGACTCTCTCCCT
IL-8	CAAGGCTGGTCCATGCTCC	TGCTATCACTTCCTTTCTGTTGC
TNF-α	GACGTGGAACTGGCAGAAGAG	TTGGTGGTTTGTGAGTGTGAG

### Western blotting

Cells were treated by RIPA lysis buffer (Beyotime Institute of Biotechnology, China) supplemented with 1% protease and phosphatase inhibitors (Beyotime, China). Then, a bicinchoninic assay (Beyotime, China) was used to measure protein concentration. Protein samples were added to the loading buffer (loading buffer to sample ratio, 1:4) and were heated to 99 °C for 10 min to denature the proteins. Eight percent or 10% sodium dodecyl sulfate-polyacrylamide gel electrophoresis (Beyotime, China) was used to separate proteins, followed by transferring to polyvinylidene fluoride membranes (Millipore, USA) and by blocking with 5% non-fat milk (BD Biosciences, USA) for 1 h at room temperature (RT). The blot was incubated with primary antibodies (1:1,000 dilutions) overnight at 4 °C (rabbit antibodies against MMP3, MMP9 , MMP13, IL-6, aggrecan, p38, phosphorylated (p)-p38, and GAPDH (Affinity Biosciences, USA); rabbit antibodies against ERK, p-ERK, JNK, p-JNK, p65, p-p65 (Cell signaling Technology, USA), and Col-2 (1:1000; Bioss, China); and mouse antibodies against Vangl2 (1:400; Santa Cruz, USA)). Then, the blots were washed for 5 min 5 times by phosphate-buffered saline supplemented with 0.05% Tween 20 (TBST) at RT. Then, they were incubated with a horseradish peroxide-conjugated goat anti-rabbit or anti-mouse IgG (1:2000; Cell Signaling Technology, USA) for 1 h (RT). An ECL Kit (Millipore, USA) was used to visualize the protein blots and Image J (version: 1.52 t; National Institutes of Health) was used for semi-quantitative analysis.

### Cell immunocytofluorescence (IF)

5000 ATDC5 were seeded in a 35-mm dish for confocal laser-scanning microscopy (MatTek Corporation, USA). Cells were treated after 24 h and then the culture medium was discarded. After being washed with PBS three times, the cells were fixed with 4% paraformaldehyde (Beyotime, China) for 15 min (RT). Subsequently, the cells were permeabilized with 0.1% Triton X-100 and blocked in 5% bovine serum albumin (BSA) for 1 h. Mouse anti-Vangl2 (1:400, Santa Cruz, USA) antibody was added to the dishes for incubation at 4 °C overnight. Cells were incubated in the dark for 1 h with secondary antibodies (488 DyLight goat anti-mouse IgG; 1:100; EarthOx, USA). The nuclei were counterstained with 4′,6-diamidino-2-phenylindole (DAPI; Beyotime, China) for 5 min after being washed with PBS. Then, the cells were observed under a fluorescence microscope (Carl Zeiss, Germany).

### Immunocytochemical (ICC) staining

The transfected ATDC5 were grown on glass coverslips that were previously coated with poly L-lysin. Then, they were fixed in 4% paraformaldehyde (Beyotime, China) for 15 min (RT). Permealization was done by immersing slides in 0.3% Triton-X100 (MP Biomedicals, USA) for 15 min. Two drops of the rabbit primary antibody against Col-2 (1:400) were applied to each slide. Then, the samples were incubated at 4 °C overnight. The next day, the Horseradish peroxidase-conjugated secondary antibody (goat anti-rabbit) was applied to each slide for 1 h. Subsequently, DAB (Zhongshan Jinqiao Biotechnology, China) was prepared and was used for visualizing any antigen-antibody reaction in the cells. The stained sections were observed and recorded by a light microscope (Leica, German).

### Statistical analysis

All statistical calculations were performed using SPSS 25.0 (IBM, USA) and each assay was performed with at least three technical and biological replicates. Normally distributed data were expressed as means ± standard deviations. Students *t* test was used to analyze the differences between the two groups. Significant differences between more than two groups were determined by the analysis of variance (ANOVA). *P* < 0.05 was considered to indicate a statistically significant difference.

## Results

### The expression of Vangl2, Wnt5a, and MMPs increased after IL-1β stimulation

**Fig. 1:**
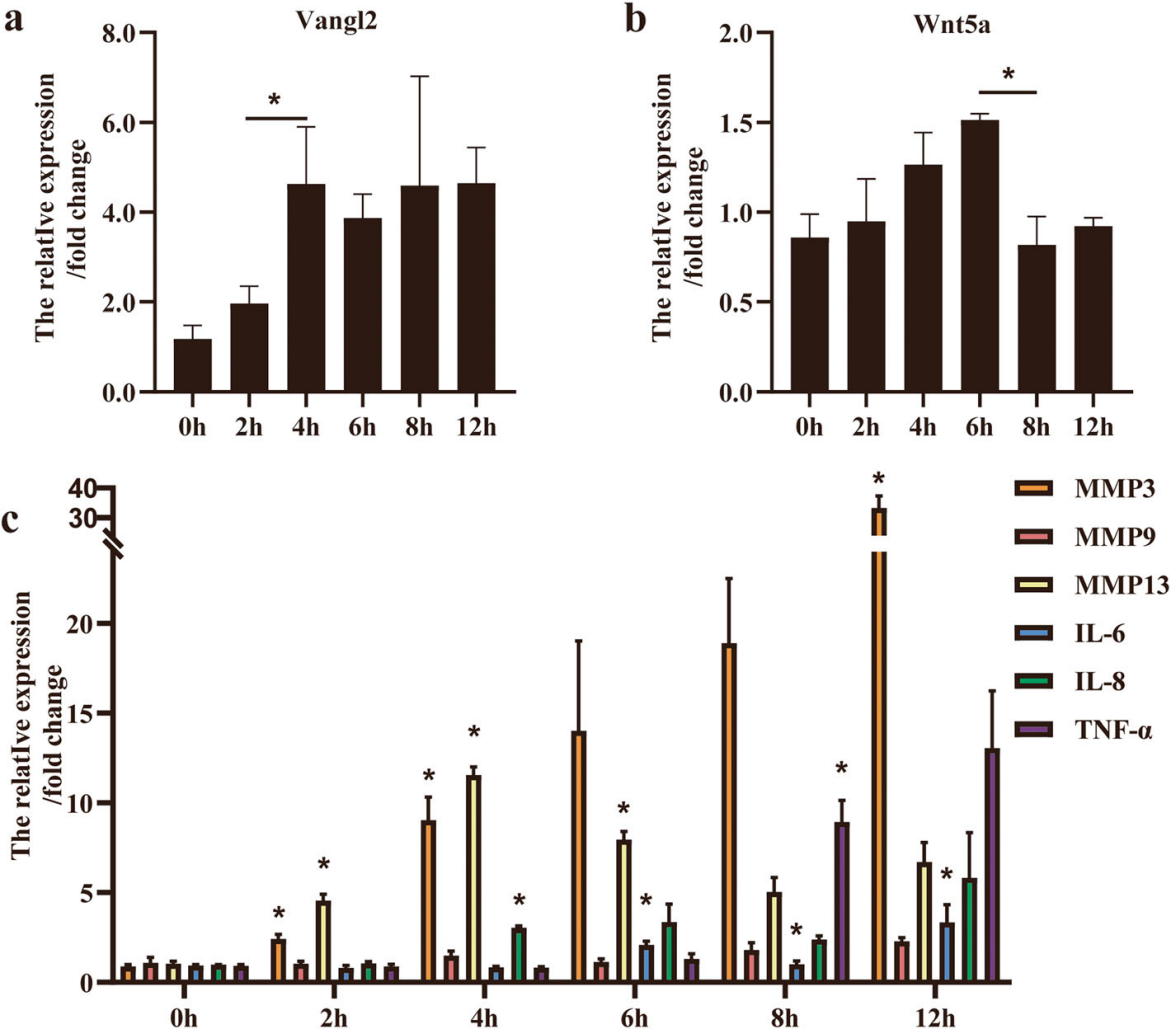
The expression of Vangl2, Wnt5a, MMPs, and proinflammatory cytokines were upregulated in OA chondrocyte. **a**, **b** Vangl2 and Wnt5a expression in ATDC5 were detected by RT-qPCR after stimulation with 10 ng/mL IL-1β for 0, 2, 4, 6, 8, and 12 h. **c** Expression trend of MMP3, MMP9, MMP13, IL-6, IL-8, and TNF-α after IL-1β stimulation were determined by RT-qPCR. *P < 0.05

Exogenous IL-1β (10 ng/mL) enhanced the mRNA expression of Vangl2, Wnt5a, MMPs, and related proinflammatory factors in a time-dependent manner. The time-course study showed that the expression of Vangl2, MMP3, and MMP13 increased at early stages. Vangl2 reached its peak at 4 h and remained at a high level for the rest of period (Fig. 1a). The expression of Wnt5a gradually elevated after IL-1β treatment and then decreased after 6 h (Fig. 1b). Although differences of MMP9 expression between adjacent time points were not statistically significant, all mRNA levels of MMP3, MMP9, MMP13, IL-6, IL-8, and TNF-α increased in IL-1β-treated chondrocyte (Fig. 1c). The expression of MMP3 changed most significantly after IL-1β stimulation and almost reached at 35 times of the initial level at 12 h. MMP13 expression also increased around 12 times of the initial value at 4 h.

**Fig. 2:**
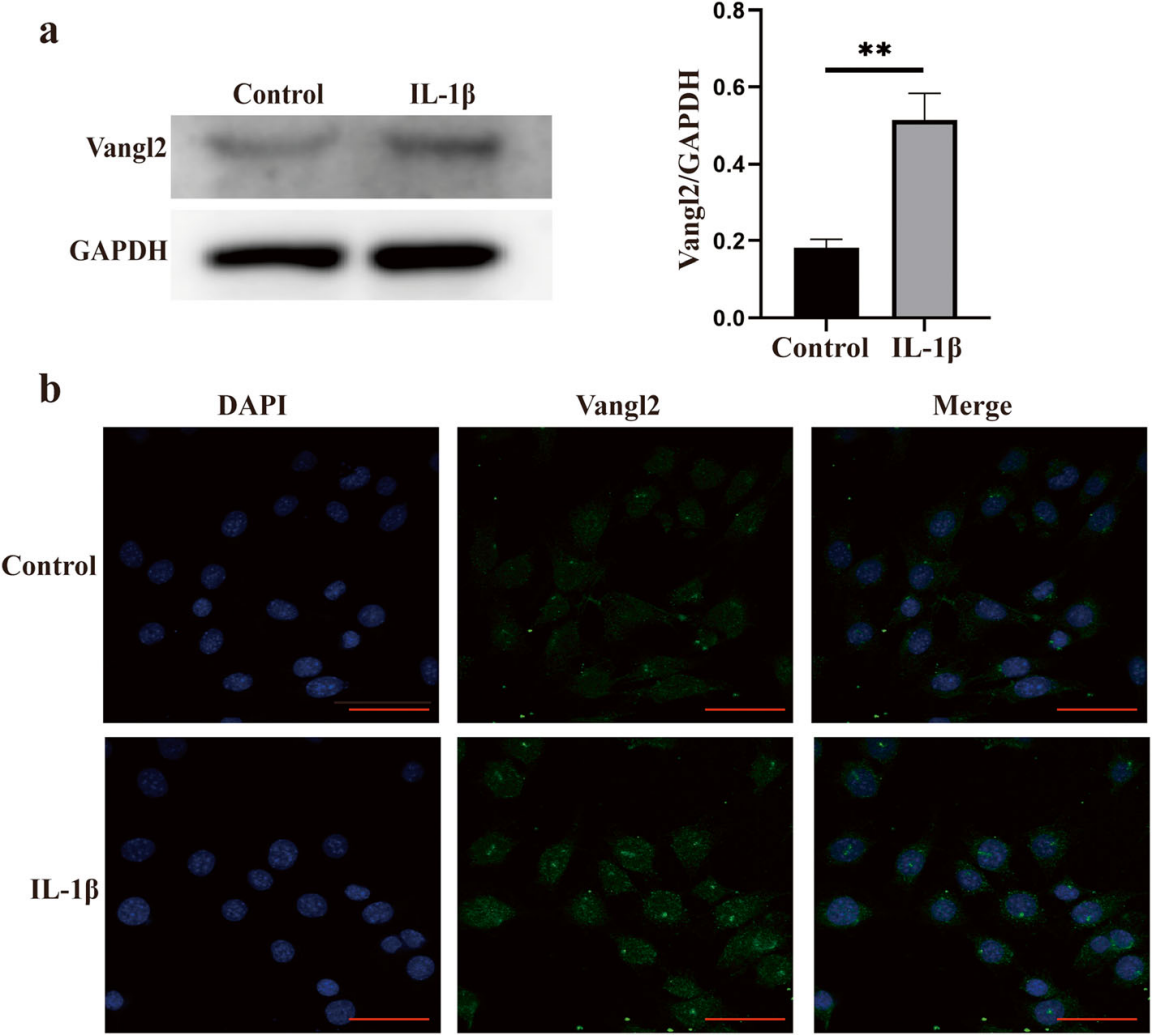
Vangl2 expression was upregulated in OA chondrocytes. **a** The protein levels of Vangl2 in normal and OA chondrocytes were detected by Western blotting and then quantified. **P < 0.01 compared with the control group. **b** Immunofluorescence for Vangl2 (green) and DAPI (blue) in control and IL-1β-treated ATDC5. Scale bar, 50 μm

Induction of Vangl2 protein by IL-1β was further verified in ATDC5 by Western blotting and IF analysis (Fig. 2a, b). A significant increase of Vangl2 immunostaining was observed after stimulation with IL-1β for 4 h, showing the consistent effects of both the mRNA and protein levels. Moreover, the increased level of Vangl2 in OA chondrocytes is also confirmed by IF staining (Fig. 2b).

### Knockdown of Vangl2 alleviated IL-1β-induced MMP 3, MMP9, MMP13, and IL-6 expression at both the gene and protein levels

**Fig. 3:**
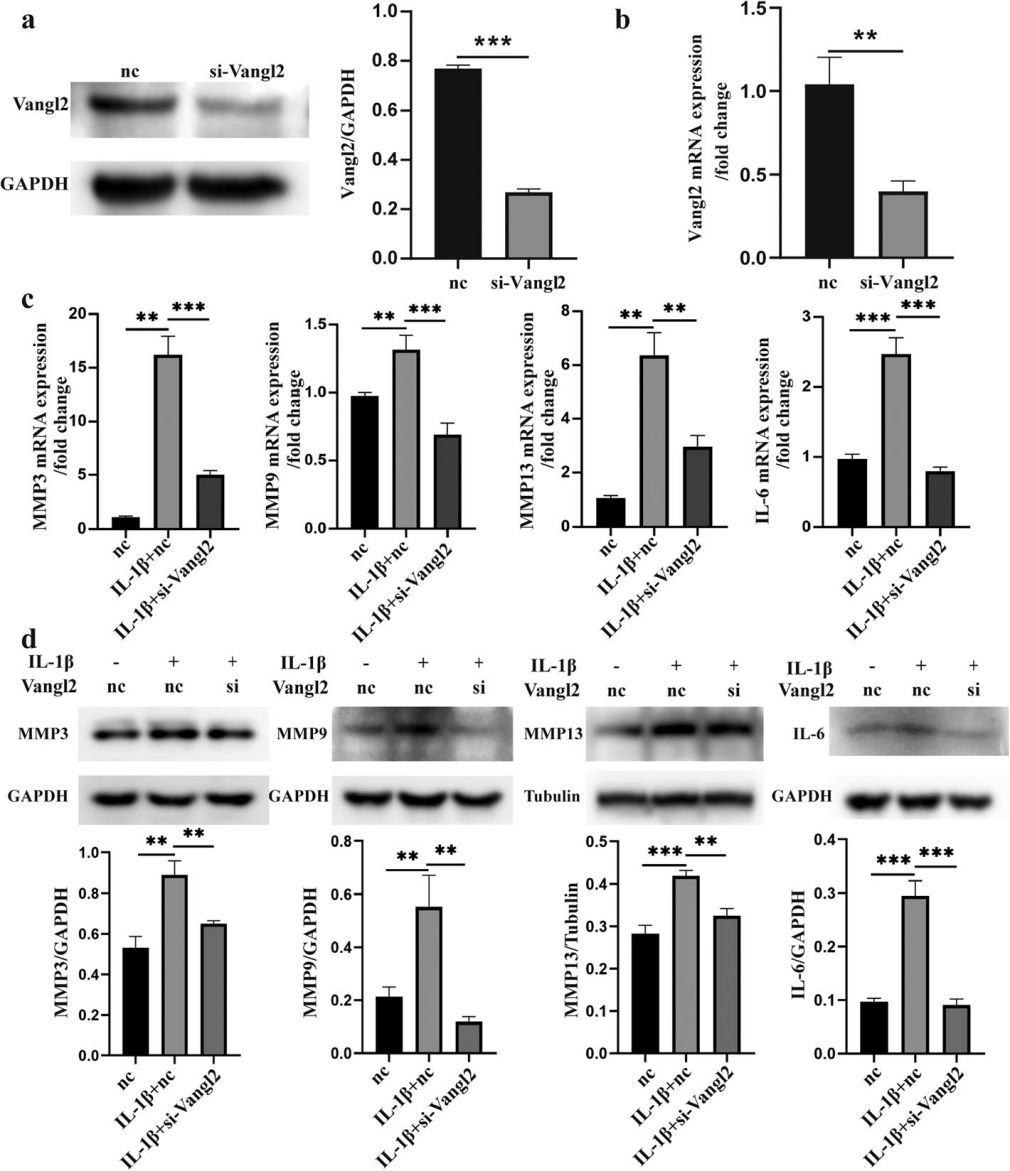
Knockdown of Vangl2 attenuated IL-1β-induced inflammatory activation in ATDC5. **a**, **b** The knockdown efficiency of siRNA targeting Vangl2 was detected by Western blotting (**a**) and RT-qPCR (**b**). **c** Effects of Vangl2 inhibition on MMP3, MMP9, MMP13, and IL-6 mRNA levels were measured by RT-qPCR. **d** Western blotting for MMP3, MMP9, MMP13, and IL-6 in three groups. Densitometric quantification values were normalized for GAPDH. **P < 0.01, ***P < 0.001

To further evaluate the effects of Vangl2 on the inflammatory response of OA chondrocyte, we analyzed whether suppression of endogenous Vangl2 modulates the expression of MMPs and IL-6. Western blotting and RT-qPCR showed that the knockdown efficiency of siRNA targeting Vangl2 was around 50% (Fig. 3a, b). The chondrocytes were divided into three groups: group I (nc), chondrocytes were only transfected by siRNA of nc; group II (IL-1β+nc), cells were further stimulated with IL-1β; and group III (IL-1β+si-Vangl2), OA chondrocytes were transfected with siRNA targeting Vangl2. The RT-qPCR and Western blotting analysis showed that gene expressions of MMP3, MMP9, MMP13, and IL-6 were significantly increased in IL-1β stimulation compared with the nc group (Fig. 3c, d). Then, we found that Vangl2 silencing notably alleviated the IL-1β-induced gene expression of MMPs and IL-6 (Fig. 3c). Western blotting also revealed that knockdown of Vangl2 reduced the protein expression of MMP3, MMP9, MMP13, and IL-6 significantly (Fig. 3d).

### Vangl2 silencing reduces the loss of cartilage ECM in vitro

**Fig. 4:**
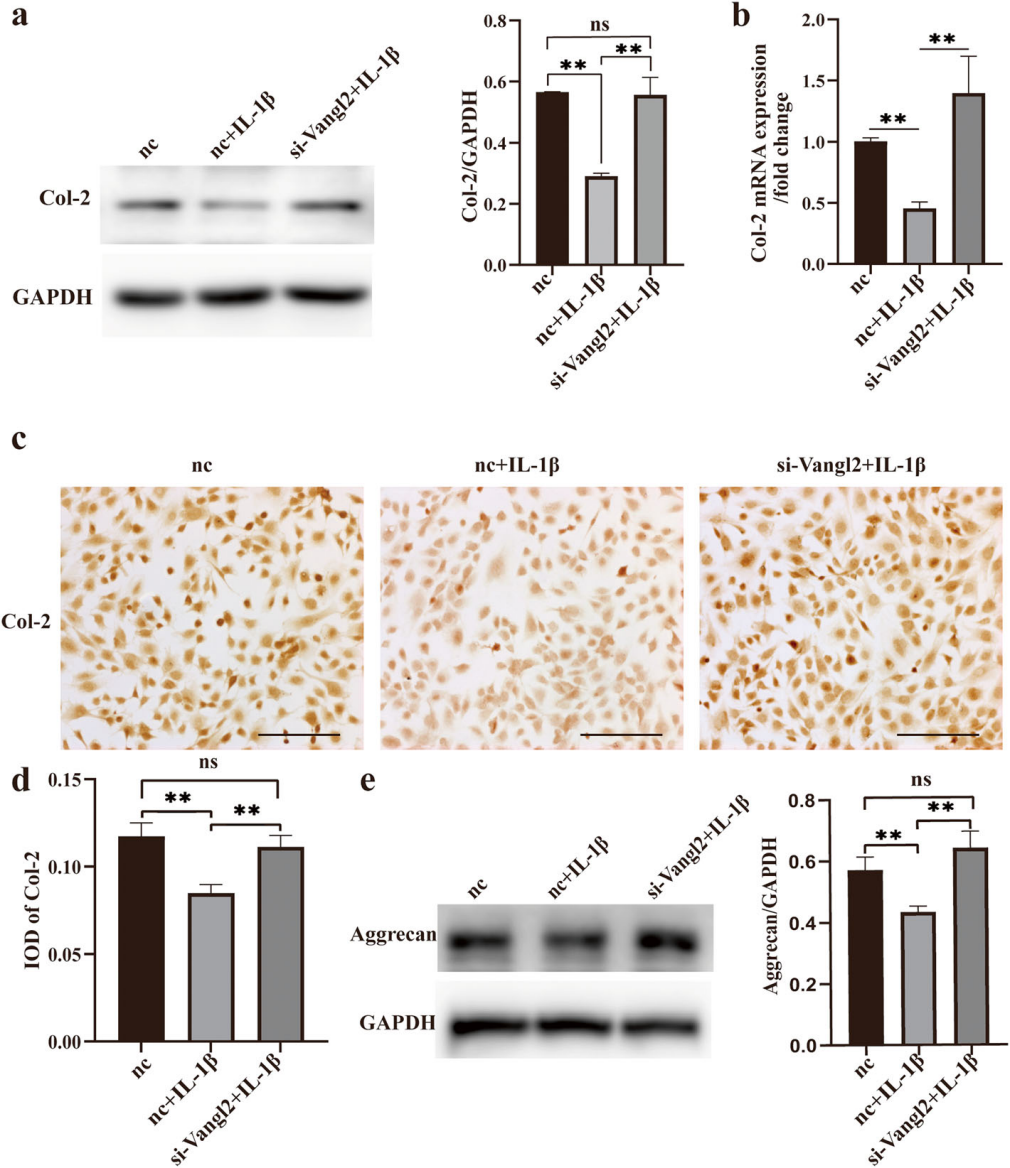
Silencing of Vangl2 prevents Col-2 and aggrecan degradation in OA. **a** Western blotting of Col-2 in three groups. The images were quantitatively analyzed and normalized to GAPDH. **b** The change of Col-2 mRNA was quantitated by RT-qPCR. Representative ICC images of Col-2 (**c**) and the IOD analysis (**d**). Scale bar, 50 μm. **e** The aggrecan expression was measured by Western blotting and quantified for three groups. ns, not statistically significant, *P < 0.05, **P < 0.01; IOD, integrated option density

To explore the relationship between Vangl2 and chondrocyte anabolism in OA, we analyzed the expression of the major structural proteins in cartilage ECM, Col-2, and aggrecan (Fig. 4). As shown in the Fig. 4, the protein and gene levels of Col-2 declined to approximately 50% after stimulated with IL-1β (Fig. 4a, b). It is of note that Vangl2 inhibition almost eliminates the effects of IL-1β on Col-2 and aggrecan. Both Western blotting and RT-qPCR showed that the expression levels of Col-2 in group III returned to normal (Fig. 4a, b). The results of ICC staining further confirmed our findings (Fig. 4c, d). Similarly, Vangl2 silencing also notably suppressed the degradation of aggrecan in OA chondrocytes (Fig. 4e).

### Inhibition of Vangl2 attenuates the proinflammatory functions of Wnt5a and the underlying mechanism

**Fig. 5:**
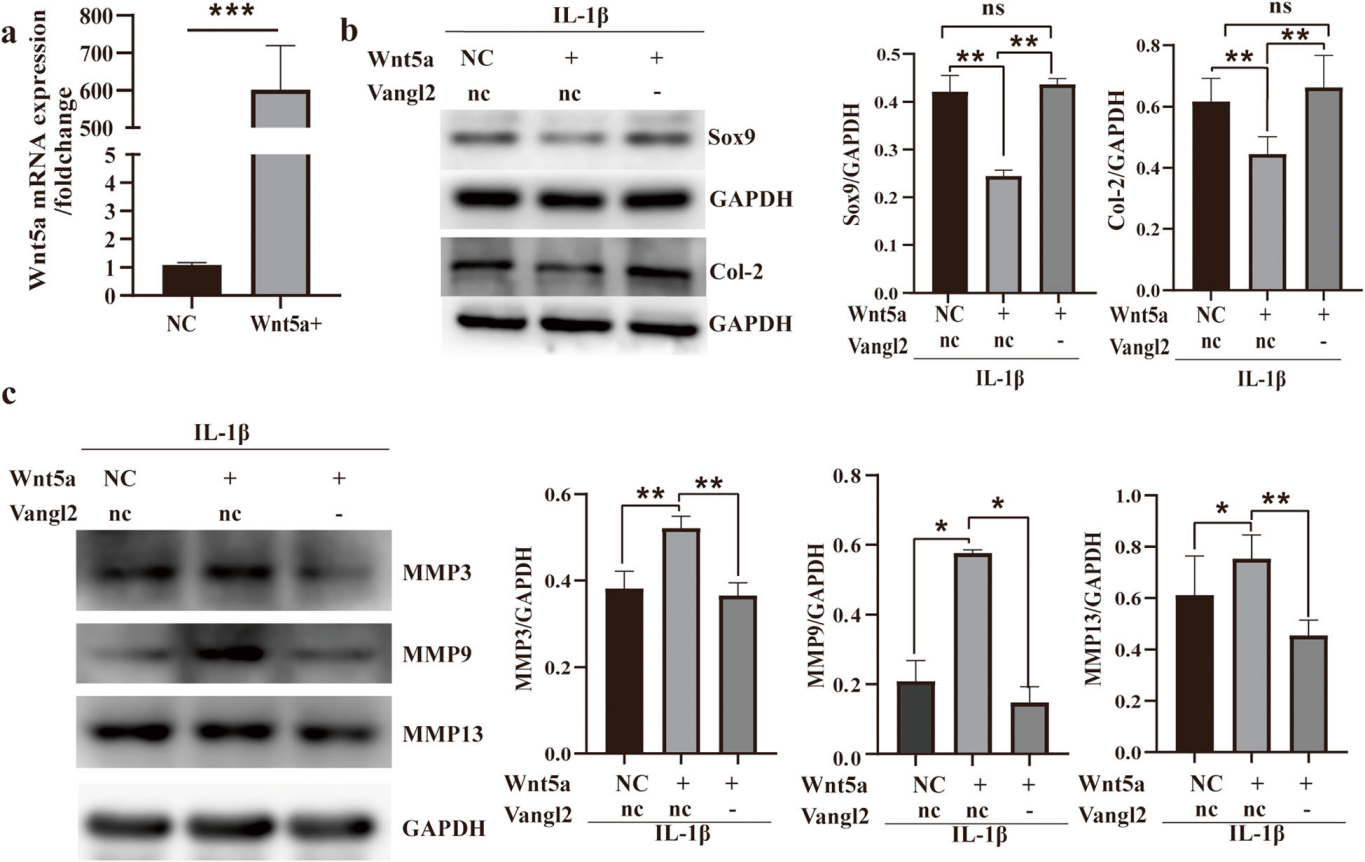
Vangl2 silencing can eliminate the effect of Wnt5a on inflammatory activation. **a** RT-qPCR analysis shows Wnt5a-overexpression efficiency of Wnt5a plasmid in ATDC5. **b** Effects of Vangl2 and Wnt5a on the expression of Sox9 and Col-2 were measured by Western blotting. **c** Protein expression of MMP3, MMP9, and MMP13 were also measured by Western blotting. GAPDH was used as a loading control. ns, not statistically significant, *P < 0.05, **P < 0.01; nc/NC, negative control; +, overexpression; -, silencing

We constructed Wnt5a-overexpressing chondrocytes by plasmid transfection and investigated the role of Wnt5a in the IL-1β-induced inflammatory response of chondrocytes (Fig. 5a). Wnt5a promoted the reduction of Sox9, Col-2, and the upregulation of MMP3, MMP9, MMP13 in OA (Fig. 5b, c). As expected, Vangl2 silencing almost eliminated the proinflammatory effects of Wnt5a in OA chondrocyte (Fig. 5b, c). Specifically, the expression of Sox9 and Col-2 was upregulated and the expression of MMP3, MMP9, and MMP13 was reduced in Vangl2-silencing chondrocyte.

**Fig. 6:**
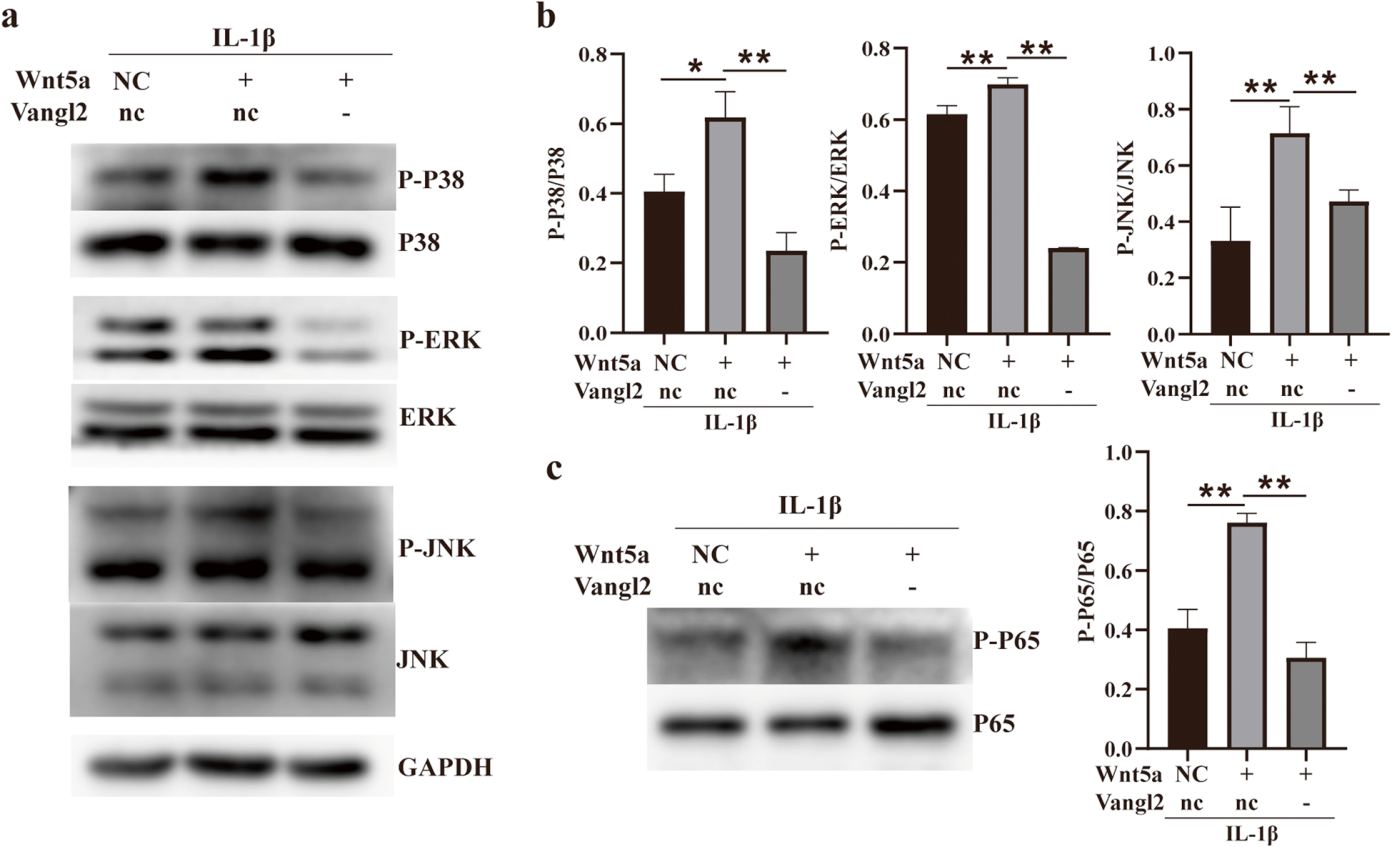
Chondrocyte signaling in response to Wnt5a and Vangl2. **a** The influence of Wnt5a and Vangl2 on MAPK signaling was detected by Western blotting. **b** MAPK signaling activation is quantified by the ratio of phosphorylated protein to total protein. **c** The effect of Wnt5a and Vangl2 on NF-κB signaling was also detected by Western blotting. *P < 0.05, **P < 0.01

To further investigate the mechanisms by which Vangl2 silencing protects chondrocytes, we examined the phosphorylation levels of P38 MAPK, extracellular signal-regulated kinase (ERK), c-Jun NH2-terminal kinase (JNK), and P65 by Western blotting (Fig. 6). The results showed that Wnt5a further upregulated the expression of phosphorylated-P38, JNK, ERK, and P65, which had been induced by IL-1β (Fig. 6a, c). Knockdown of Vangl2 significantly inhibited the expression of P-P38, P-JNK, and P-ERK induced by Wnt5a (Fig. 6a, b). Simultaneously, Vangl2 silencing also notably decreased the phosphorylation level of P65 enhanced by Wnt5a (Fig. 6c).

## Discussion

Cellular functions of Vangl2 have been explored for more than two decades since Vangl2/Stbm was first identified in *Drosophila* [24]. A previous study in human revealed that the expression of Vangl2 increased in the OA synovium [17]. Thus, our finding that Vangl2 was significantly increased in OA chondrocytes raised the question of whether Vangl2 plays a role in OA progression. In this study, we demonstrate for the first time that Vangl2 is involved in OA progression, and Vangl2 silencing can suppress the proinflammatory functions of Wnt5a in OA chondrocytes.

In our study, the upregulation of Vangl2 was determined at an early stage during IL-1β stimulation, which is similar with MMPs. This finding suggests a link between Vangl2 and MMPs, the key mediators of the cartilage destruction in OA [25]. Our further experiments provide evidence that knockdown of Vangl2 corrected the imbalance between anabolic and catabolic factors in IL-1β-treated chondrocytes, resulting in the inhibition of MMP3, MMP9, MMP13, and IL-6 as well as the upregulation of Col-2 and aggrecan. In this study, MMP3 and MMP13 were significantly suppressed by Vangl2 silencing. Surprisingly, we found that the main gelatinases, MMP9, decreased to the level of control group in Vangl2-silencing chondrocyte. These MMPs are major contributors to the degenerative process in OA due to their digestion of cartilage ECM [25, 26]. Among them, MMP13 can cleave the triple helix structure of Col-2, the predominant collagen in cartilage, and other MMPs can further digest the collagen hydrolysate, as well as aggrecan [27]. These results confirmed our notion that Vangl2 regulates MMPs in OA chondrocytes, which is also supported by prior findings that Vangl2 can directly regulate MMPs in other cells [28, 29]. Moreover, Vangl2 silencing restored the expression of Col-2 and aggrecan, which are the main structural proteins of cartilage ECM. Collectively, our results indicate that Vangl2 silencing protects chondrocyte from IL-1β-induced metabolic disorder in vitro.

Unexpectedly, we found that Vangl2 silencing almost eliminates the proinflammatory effects of Wnt5a in OA chondrocytes in this experiment. The increased expression of MMP3, MMP9, and MMP13 by Wnt5a was significantly suppressed by the inhibition of Vangl2. Notably, the expression of Sox9, a key transcription factor related to cartilage formation, and Col-2, returned to the baseline levels after Vangl2 silencing. Our finding that Wnt5a promotes inflammation via multiple pathways in OA chondrocytes is consistent with the results reported before [9, 30, 31]. These data show that all three MAPK pathways (p38, ERK, JNK) can be activated by Wnt5a in OA chondrocytes. The MAPK pathway is one of the most important signal transduction systems in OA pathogenesis, which regulates cartilage ECM degradation (MMPs production) and synthesis (Sox9/Col-2 expression) [32–34]. Furthermore, we observed a marked upregulation of p-p65 (the representative protein of NF-κB pathway) in Wnt5a-overexpressing chondrocytes. NF-κB is an essential transcription factor that, together with other pathways including the MAPK pathway, mediates inflammation and regulates catabolism and apoptosis of chondrocytes in OA [35–37]. More importantly, we found that Vangl2 knockdown remarkably decreased the activation of MAPK and NF-κB pathway that induced by Wnt5a in this study. In general, our results reveal that Vangl2 silencing notably suppresses the proinflammatory functions of Wnt5a via the MAPK and NF-κB pathway in OA chondrocytes. Increasing our knowledge of Vangl2 may provide new opportunities for the development of OA treatment.

## Conclusion

For the first time, we demonstrate that Vangl2 is involved in the OA process. Vangl2 silencing can notably alleviate OA progression in vitro by correcting the imbalance between anabolic and catabolic factors and can inhibit the proinflammatory effects of Wnt5a via MAPK and NF-κB pathway in IL-1β-treated chondrocytes. This study provides new insight into the mechanisms of cartilage inflammation.

## Data Availability

We state that the data will not be shared since all the raw data are present in the figures included in the article.
